# Morphologic and Hemodynamic Analysis in the Patients with Multiple Intracranial Aneurysms: Ruptured versus Unruptured

**DOI:** 10.1371/journal.pone.0132494

**Published:** 2015-07-06

**Authors:** Linkai Jing, Jixing Fan, Yang Wang, Haiyun Li, Shengzhang Wang, Xinjian Yang, Ying Zhang

**Affiliations:** 1 Department of Interventional Neuroradiology, Beijing Neurosurgical Institute, Beijing Tiantan Hospital, Capital Medical University, Beijing, China; 2 Department of Biomedical Engineering, Capital Medical University, Beijing, China; 3 Department of Mechanics and Engineering Science, Fudan University, Shanghai, China; Geisinger Clinic, UNITED STATES

## Abstract

**Background and Purpose:**

The authors evaluated the impact of morphologic and hemodynamic factors on multiple intracranial aneurysms and aimed to identify which parameters can be reliable indexes as one aneurysm ruptured, and the others did not.

**Methods:**

Between June 2011 and May 2014, 69 patients harboring multiple intracranial aneurysms (69 ruptured and 86 unruptured) were analyzed from 3D-digital subtraction angiography (DSA) images and computational fluid dynamics (CFD). Morphologic and hemodynamic parameters were evaluated for significance with respect to rupture. Receiver operating characteristic (ROC) analysis identified area under the curve (AUC) and optimal thresholds separating ruptured from unruptured intracranial aneurysms for each parameter. Significant parameters were examined by binary logistic regression analysis to identify independent discriminators.

**Results:**

Nine morphologic (size, neck width, surface area, volume, diameter of parent arteries, aspect ratio, size ratio, lateral/bifurcation type and regular/irregular type) and 6 hemodynamic (WSSmean, WSSmin, OSI, LSA, flow stability and flow complexity) parameters achieved statistical significance (*p*<0.05). Six morphologic (size, surface area, volume, aspect ratio, size ratio and regular/irregular type) and five hemodynamic (WSSmean, WSSmin, LSA, flow stability and flow complexity) parameters had high AUC values (AUC>0.7). By binary logistic regression analysis, large aspect ratio and low WSSmean were the independently significant rupture factors (AUC, 0.924; 95% CI, 0.883–0.965).

**Conclusions:**

Large aspect ratio and low WSSmean were independently associated with the rupture status of multiple intracranial aneurysms.

## Introduction

Intracranial aneurysms are pathological dilatations of the cerebral arteries, which are present in 2%–5% of the general population and the annual risk of rupture was 0.7%–1.9%, causing subarachnoid hemorrhage (SAH) [1]. Approximately, the incidence of multiple intracranial aneurysms varies from 15% to 35% presenting with SAH [2–6]. In patients with multiple intracranial aneurysms suffering from SAH, it is of vital importance to determine which aneurysm is the cause of SAH if all the aneurysms cannot be treated at a time, either by endovascular coiling or surgical clipping [7–10]. For patients without SAH, identifying the aneurysms in danger of rupture before operation is valuable for preventive treatment too. The clinical misdiagnosis is dangerous because the untreated but ruptured aneurysm may rupture again very soon [9, 10].

The rupture risk assessments for multiple intracranial aneurysms are mainly based on morphology [4, 5, 7, 8, 11, 12]. Hemodynamics play a fundamental role in aneurismal rupture and computational fluid dynamics (CFD) has become a popular tool for studying hemodynamics [13–18]. It is largely unknown whether hemodynamic factors are also involved in modulating the risk of rupture in multiple intracranial aneurysms. A comparison of morphologic and hemodynamic factors between ruptured and unruptured multiple intracranial aneurysms is therefore of great value, since the findings may provide an important reference for neurosurgeons.

We have performed a retrospective study of patients with multiple intracranial aneurysms who were managed at our institution over the past 36 months. Our aim was to explore the reliable indexes associated with aneurismal rupture on the basis of the morphologic and hemodynamic characteristics of multiple intracranial aneurysms.

## Methods

### Patient Selection

The retrospective study was approved by Beijing Tiantan Hospital's ethics committee and written informed consents were obtained from patients or their family members. During a 3-year period (June 2011 to May 2014), 69 consecutive patients, suffering SAH, with 155 saccular aneurysms (69 ruptured, 86 unruptured) were diagnosed and treated by either coiling or clipping in our institute. We divided aneurysms into two groups, ruptured and unruptured, based on their different rupture status and performed a retrospective analysis. The inclusion and exclusion criteria were as follows:

The inclusion criteria were: (1) multiple intracranial saccular aneurysms with different rupture status in the same patient; (2) the ruptured aneurysm was identified by intraoperative findings or head CT scan imaging; (3) 3D-DSA images and complete clinical data were of adequate resolution for CFD analysis. The exclusion criteria were: (1) fusiform or dissecting aneurysms; (2) the ruptured aneurysm was not identified; (3) incomplete 3D-DSA images or clinical data were not of adequate resolution for CFD analysis.

### Hemodynamics Models

Briefly, patient-specific 3D-DSA data were obtained and software package developed in-house was used to create and modify a stereolithographic image that contained the blood vessel luminal surface information [19, 20]. The aneurysm and the 10 mm of vessel surrounding it were separated for the analysis [21]. Each model was merged in the ICEM CFD software (ANSYS, Inc., USA) to create more than one million finite volume tetrahedral elements grids. The grid-dependency on the average value of hemodynamic factors has been confirmed and the maximum element size was set to 0.2 mm [22]. After meshing, ANSYS CFX 14.0 software (ANSYS, Inc., USA) was used for simulation of hemodynamics. The governing equations underlying the calculation were the Navier–Stokes formulation, with an assumption of a homogenous, laminar and incompressible blood flow. We treated blood as a Newtonian fluid. The blood vessel wall was assumed to be rigid with no-slip boundary conditions. The density and dynamic viscosity of blood were specified as ρ = 1060 kg/m^3^ and μ = 0.004Pa・s, respectively. A representative pulsatile period velocity profile was obtained by transcranial Doppler and set as the inflow boundary condition. Two cardiac cycle simulations were performed for numerical stability. To confirm the numeric stability, the results from the second cardiac cycle were collected as output for the final analyses. The average Reynolds number was within the range of normal blood flow in human cerebral arteries.

### Data Collection and Data Analysis

Morphologic parameters included size (length from the neck center to the dome of the aneurysm), neck width (the average orifice diameter), surface area, volume, diameter of parent artery, aspect ratio (dome-to-neck ratio) and size ratio (dome-to-parent artery diameter ratio) were measured and calculated from 3D-DSA data. The number of each morphologic types such as lateral / bifurcation (aneurysms originating from only one parent vessel or from the origin of a small branch whose caliber is less than one fifth of the parent vessel are classified as lateral aneurysms; otherwise, bifurcation aneurysms)[17, 23] and regular / irregular (An aneurysm was defined as being irregularly shaped when blebs, aneurysm wall protrusions, or multiple lobes were present)[8] were recorded.

After CFD simulation, the following hemodynamic factors in the two groups were calculated and compared: (1) Wall shear stress (WSS) related factors: the time-averaged WSS was calculated by integrating the WSS magnitude at each node over the cardiac cycle. The time-averaged WSS was normalized by the average parent vessel WSS in the same patient to minimizes the dependence on inlet conditions [21]. Then the mean, maximum WSS (WSSmax) and minimum WSS (WSSmin) were recorded. (2) The mean Oscillatory shear index (OSI) value: $OSI=\frac{1}{2}\left(1-\frac{\left|\int_{0}^{T}wss_{i}dt\right|}{\int_{0}^{T}\left|wss_{i}\right|dt}\right)$ where *wss*_*i*_ is the instantaneous WSS vector and T is the duration of the cycle [24]. (3) Low wall shear area (LSA): LSA was defined as the areas of the aneurysm wall exposed to a WSS below 10% of the mean surrounding vessel WSS and then normalized by the dome area [25]. (4) Flow pattern: flow stability (the stable flow pattern persisted, while the unstable flow pattern had flow structure moved or changed during the cardiac cycle) and flow complexity (the simple flow pattern had a single vortex structure, while the complex flow contained multiple vortices) [26].

Univariate analyses were performed to get valuable parameters: for quantitative data, the one-sample Kolmogorov–Smirnov test was used to test the normal distribution, then independent sample t-test was used for all the approximately normal distributed parameters with data expressed as mean ± SD, the Rank-sum test was used for nonnormally distributed parameters with data expressed as median (quartile). For qualitative data, the chi-square test was performed. The area under the receiver operating characteristics (ROC) curve (AUC) and the optimal thresholds using the Youden index was calculated from ROC analysis. The binary logistic regression (backward elimination) was performed to assess the independent relationship of all significant univariate factors with aneurismal ruptures. The odds ratio (OR) and 95% confidence interval (CI) of the results were obtained. And the model’s calibration was evaluated using the Hosmer and Lemeshow Test. Statistical analysis was performed with an SPSS 17.0 package. *p*<0.05 was regarded as statistically significant.

## Results

### Clinical Characteristics

69 patients aged between 28 and 84 years (mean 60.1y) were analyzed, of which 55 (79.7%) were females and 14 (20.3%) were males. Totally 155 aneurysms were found in these patients, of which 69 were ruptured and 86 were unruptured. The most common sites of aneurysms were the ICA and MCA (98/155 and 36/155, respectively), followed by the ACoA (12/155). Aneurysms located on the ACoA had the highest rupture ratio (10/12). (S1 Table)

### Morphologic Factors

**Fig 1:**
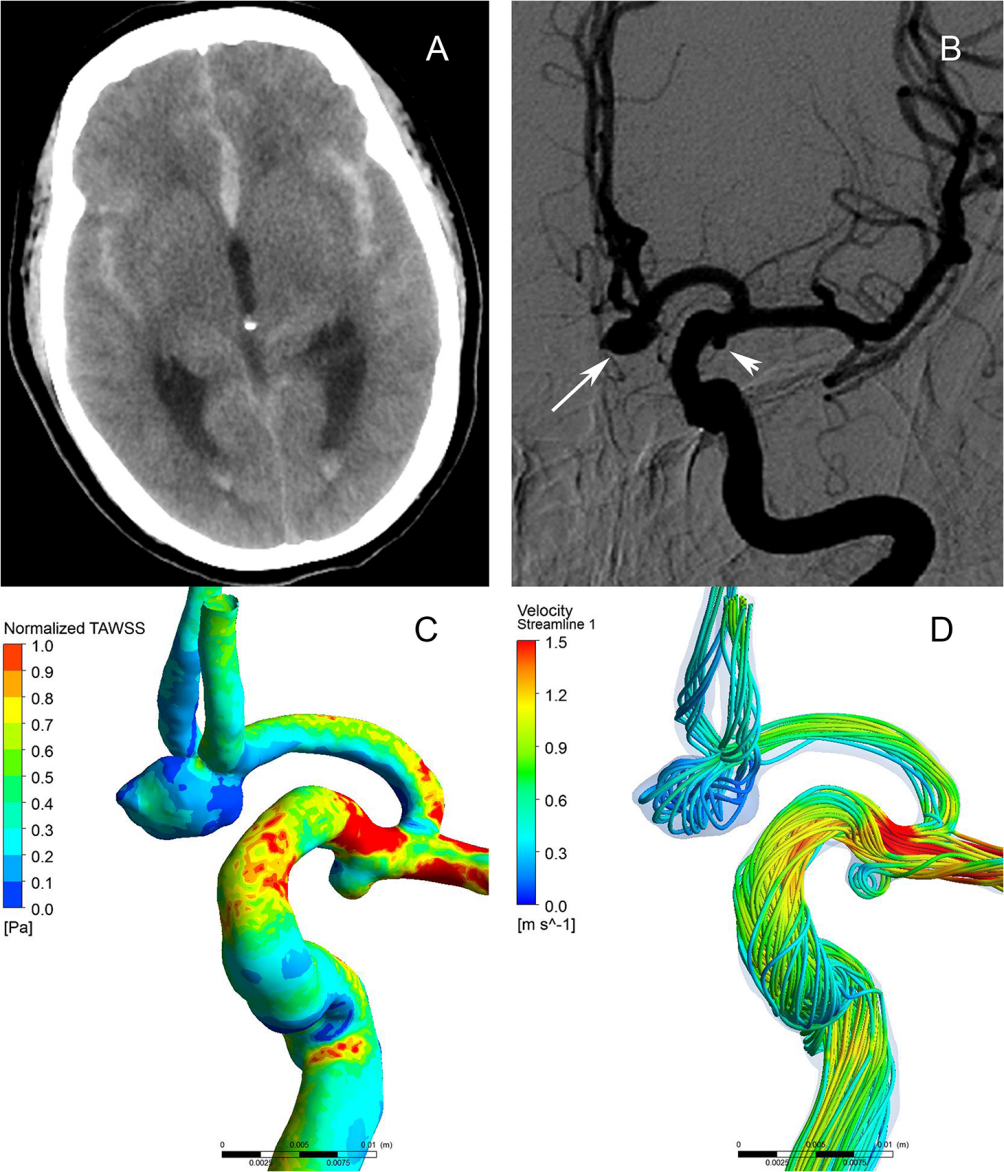
Morphological-hemodynamic characteristics of multiple intracranial aneurysms in the ipsilateral anterior circulation. A 60-year-old female presented with severe headache. CT scan showed subarachnoid hemorrhage (A). The angiograms of left internal carotid artery showed a ruptured aneurysm (B, large arrow) and an unruptured aneurysm (B, small arrow). The distribution of normalized wall shear stress (WSS) magnitude on the aneurysms sac showing the ruptured aneurysm had a lower WSS than the unruptured one (C). The Streamlines showing the flow pattern of the ruptured aneurysm was complex at peak systole (D). Streamlines are rendered with colors according to the velocity magnitude.

**Fig 2:**
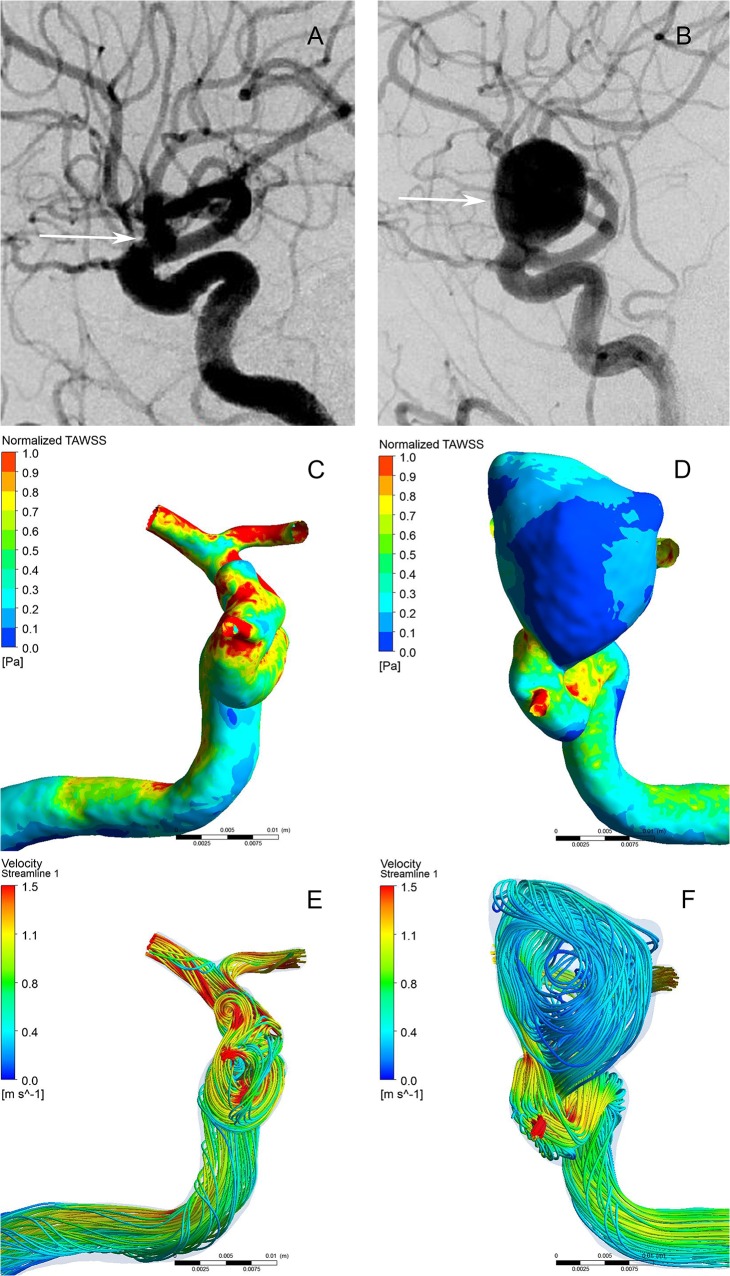
Morphological-hemodynamic characteristics of intracranial mirror aneurysms. A 42-year-old male presented with severe headache. The angiograms of internal carotid artery showed an unruptured aneurysm (A) and a ruptured aneurysm (B). The distribution of normalized WSS magnitude on the aneurysms sac showing the ruptured aneurysm had a lower WSS than the unruptured one (C and D). The Streamlines showing the flow pattern of the ruptured aneurysm was complex at peak systole (E and F). Streamlines are rendered with colors according to the velocity magnitude.

As present in Tables 1 and 2, the ruptured aneurysms had significantly larger size (*p*<0.001), neck width (*p* = 0.017<0.05), surface area (*p*<0.001), volume (*p*<0.001), aspect ratio (*p*<0.001), size ratio (*p*<0.001) but smaller parent artery diameter (*p* = 0.041<0.05) than the unruptured aneurysms. (Fig 1B and Fig 2A and 2B) The ruptured aneurysms usually had irregular shapes (*p*<0.001) and located at bifurcation (*p* = 0.01).

**Table 1:** Result from Univariate statistical analysis and ROC analysis for all quantitative variables.

Variables	Aneurysms				ROC analysis		
	Total	Ruptured	Unruptured	*p* value*	AUC (95%CI)	Threshold	*p* value^#^
	(n = 155)	(n = 69)	(n = 86)				
Size, mm	3.81 (3.508)	5.55 (3.261)	2.84 (1.345)	<0.001	0.879 (0.824–0.934)	4.217	<0.001
Neck width, mm	3.84 (1.320)	4.14 (1.529)	3.61 (1.078)	0.017	0.591 (0.498–0.683)	3.759	0.053
Surface area, mm^2^	38.87 (51.841)	69.71 (88.644)	27.84 (23.316)	<0.001	0.811 (0.742–0.879)	38.83	<0.001
Volume,mm^3^	27.55 (57.591)	60.41 (114.450)	16.45 (20.497)	<0.001	0.785 (0.712–0.857)	27.08	<0.001
Parent artery diameter, mm	3.24 (0.985)	3.06 (0.881)	3.38 (1.044)	0.041	0.576 (0.486–0.666)	3.807	0.106
Aspect ratio	1.17 (0.523)	1.56 (0.507)	0.86 (0.263)	<0.001	0.918 (0.876–0.961)	1.064	<0.001
Size ratio	1.32 (1.273)	1.88 (1.218)	0.84 (0.582)	<0.001	0.876 (0.822–0.929)	1.222	<0.001
WSSmean	0.69 (0.266)	0.53 (0.223)	0.83 (0.284)	<0.001	0.834 (0.768–0.900)	0.729	<0.001
WSSmax	3.04 (0.757)	3.04 (0.822)	3.04 (0.706)	0.984	0.519 (0.426–0.613)	2.894	0.679
WSSmin	0.02 (0.037)	0.01 (0.013)	0.04 (0.063)	<0.001	0.799 (0.730–0.868)	0.023	<0.001
OSI	0.0075 (0.017)	0.0109 (0.024)	0.0058 (0.010)	<0.001	0.671 (0.587–0.756)	0.005	<0.001
LSA, %	0.65 (6.255)	3.06 (13.990)	0.07 (0.593)	<0.001	0.836 (0.774–0.898)	0.599	<0.001

WSS, wall shear stress; WSSmax, the maximum intra-aneurysmal WSS magnitude; WSSmin, the minimum intra-aneurysmal WSS magnitude; OSI, Oscillatory shear index; LSA, low wall shear area.

* Independent sample t test or Rank-sum test as appropriate. *p*<0.05 was considered statistically significant.

^#^ *p* values for ROC analysis and *p*<0.05 was considered statistically significant.

**Table 2:** Result from univariate statistical analysis and ROC analysis for all qualitative variables.

Variables	Aneurysms					ROC analysis	
	Total	Ruptured	Unruptured	X^2^	*p* value*	AUC (95%CI)	*p* value^#^
	(n = 155)	(n = 69)	(n = 86)				
Morphology type							
Lateral (%)	74 (47.7)	25 (36.2)	49 (57.0)	6.604	0.01	0.604 (0.514–0.693)	0.027
Bifurcation (%)	81 (52.3)	44 (63.8)	37 (43.0)				
Regular (%)	99 (63.9)	23 (33.3)	76 (88.4)	50.257	<0.001	0.775 (0.697–0.853)	<0.001
Irregular (%)	56 (36.1)	46 (66.7)	10 (11.6)				
Flow stability							
Stable (%)	101 (65.2)	27 (39.1)	74 (86.0)	37.12	<0.001	0.735 (0.652–0.817)	<0.001
Unstable (%)	54 (34.8)	42 (60.9)	12 (14.0)				
Flow complexity							
Simple (%)	98 (63.2)	26 (37.7)	72 (83.7)	34.902	<0.001	0.730 (0.648–0.813)	<0.001
Complex (%)	57 (36.8)	43 (62.3)	14 (16.3)				

* Chi-square test as appropriate. *p*<0.05 was considered statistically significant.

^#^ *p* values for ROC analysis and *p*<0.05 was considered statistically significant.

### Hemodynamic Factors

As present in Tables 1 and 2, the ruptured aneurysms had significantly lower WSSmean (*p*<0.001) and WSSmin (*p*<0.001), higher OSI (*p*<0.001) and LSA (*p*<0.001) than the unruptured aneurysms (Fig 1C and Fig 2C and 2D). In addition, ruptured aneurysms usually had complex flow patterns with multiple vortices, while unruptured aneurysms usually had simple flow patterns with a single vortex (*p*<0.001) (Fig 1D and Fig 2E and 2F). WSSmax showed no significant differences between the two groups (*p*> 0.05).

### ROC analysis and Binary Logistic Regression Analysis

The ROC-AUC values associated with rupture are displayed in Tables 1 and 2. Six morphologic (size, surface area, volume, aspect ratio, size ratio and regular/irregular type) and 5 hemodynamic (WSSmean, WSSmin, LSA, flow stability and flow complexity) parameters had high AUC values (AUC>0.7). Other factors did not perform well (AUC<0.7).

**Fig 3:**
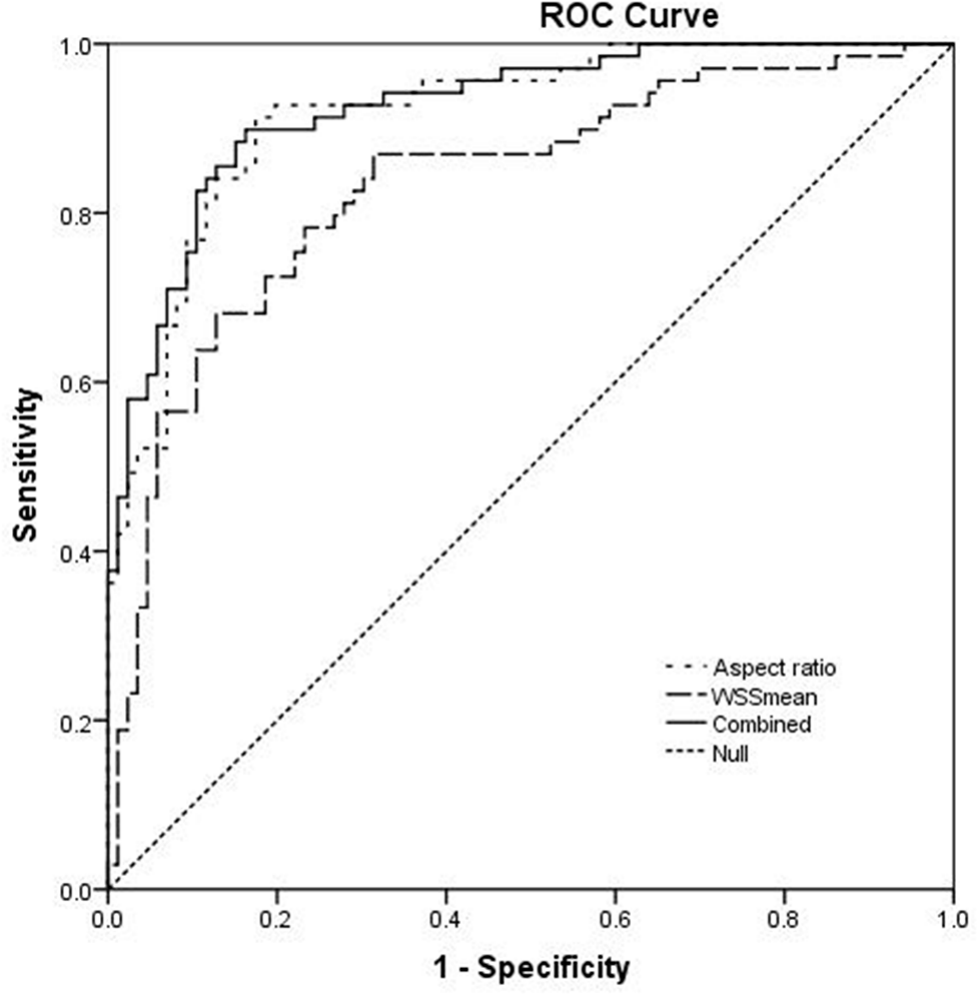
Receiver operating characteristic (ROC) curves for key parameters.

Aspect ratio and WSSmean were found to be independent predictive factors for multiple aneurismal rupture (AUC, 0.924; 95% CI, 0.883–0.965) (Table 3, Fig 3). The Hosmer-Lemeshow test showed significant agreement between predicted risk and observed outcome (*p* = 0.856>0.05).

**Table 3:** Independently significant risk factors.

Variables	OR^#^ (95% CI)	*p* value *
Aspect ratio	162.269 (24.821–1060.826)	<0.001
WSSmean	0.092 (0.009–0.947)	0.045

^#^ the adjusted odds ratio.

* Binary logistic regression analysis and p<0.05 was considered statistically significant.

## Discussion

Many studies have explored the risk factors of aneurysm rupture by comparing the difference between ruptured and unruptured solitary aneurysms, mainly based on clinical and/or morphologic date. However, patient-related genetics and environmental factors can affect the results. With the technological advancements in vascular imaging, we have attempted to explore the potential rupture risk of multiple aneurysms in the same patient through the aneurismal geometry and hemodynamic effect and identify which parameters can be reliable indexes as one aneurysm ruptured, and the others did not.

In terms of morphology, many parameters were significantly associated with the ruptured aneurysms by univariate analysis. However, after multivariate logistic regression analysis, only aspect ratio was independently significant parameter for rupture. Most but not all previous studies showed that mean aspect ratio was higher in ruptured aneurysms than unruptured [7, 8, 27–29]. Ujiie *et al*[27] produced 17 different aneurysms in rabbits and found that the localized, extremely low-flow condition that was observed in the dome of aneurysms with aspect ratio of more than 1.6 was a common flow characteristic in the geometry of ruptured aneurysms. Their further study reviewed the two-dimensional angiogram from 129 ruptured and 78 unruptured aneurysms, and found that almost 80% of the ruptured aneurysms showed a higher aspect ratio (>1.6), whereas almost 90% of the unruptured aneurysms showed a small aspect ratio (<1.6) [29]. In our studies, aspect ratio was a vital independent risk factor for multiple intracranial aneurysms. However, the 1.6 threshold value was not confirmed. The aspect ratio ≥1.064 was associated with aneurysm rupture. The reason may be that the difference of single aneurysm and multiple aneurysms, and the measurements of 2D angiogram can be more exaggerated due to an overlay effect than 3D reconstructed images. [7, 8, 29] To minimize the influence of patient-specific characteristics and images, several studies were conducted by using multiple intracranial aneurysms and 3D reconstructed images [7, 8]. Backes *et al*[8] performed conditional univariable logistic regression analysis using 3D-CTA images on 124 patients with 302 multiple aneurysms and found that aspect ratio ≥1.3 is associated with multiple aneurysm rupture independent of aneurysm size and location, and independent of patient characteristics. Using high quality 3D-DSA images, we also found that the aspect ratio, with high AUC value (AUC, 0.918; 95% CI, 0.876–0.961), seems to be the best discriminator for multiple aneurysms rupture status, consistent with finding from Jeon *et al*[7]. Aspect ratio on its own may be not a reliable variable for the prediction of aneurysm rupture, but it is an additional risk factor of which aneurysm can be assumed to have caused SAH in patients with multiple intracranial aneurysms.

The hemodynamics is thought to play an important role in the aneurysm rupture [13–18, 24–26, 30, 31]. Many studies focused on qualitative parameters, in which complex flow patterns and multiple vortices have been associated with ruptured aneurysms. From the mechanistic perspective, they speculated that complex flow patterns maybe increase inflammatory cell infiltration in the aneurysmal wall [14, 30]. In the present study, the flow stability and flow complexity also had small probability value (*P<*0.001), high AUC values (AUC>0.7), but were not retained as significance by multivariate logistic regression. However, for quantitative hemodynamic parameters, our study indicated that WSSmean was independently significant parameters for rupture by multivariate logistic regression analysis. In this study, the ruptured group had lower WSSmean than the unruptured group with a clinically useful AUC value (AUC, 0.834; 95% CI, 0.768–0.900), consistent with findings from Lu *et al*[13] and Xiang *et al*[17]. Previous studies revealed that the WSS was converted to biological signals through mechanoreceptors on endothelial cells, and then, it modulated gene expressions and the cellular functions of vessel walls [17, 32, 33]. The findings that the degeneration of aneurysm wall increases from neck to dome and aneurysm rupture mostly occurs at the dome give an indication that specific level of WSS was necessary for maintaining endothelial cell vitality and arterial integrity [32, 34–36]. Low WSS is known to upregulate endothelial surface adhesion molecules, cause dysfunction of flow-induced nitrous oxide, increase endothelial permeability, and thus, promote atherogenesis and inflammatory cell infiltration [17, 32, 33]. This atherosclerotic and inflammatory pathway triggered by these hemodynamic conditions may cause degradation of the aneurysm wall that could ultimately lead to rupture [17, 27, 29, 32]. Low WSS may be conducive for atherosclerotic change and inflammatory responses, which could drive heterogeneous remodeling of the aneurysm wall and aneurysm growth. Such growth could lead to an increasing aspect ratio. High aspect ratio values, in turn, would lead to lower WSS.

Similar to other CFD analyses, rigid wall, Newtonian blood, laminar flow and typical flow waveform on healthy subject were used during the modeling process which may be affecting the reality of the hemodynamic result. Although the study of 155 aneurysms is a small sample and biases are inevitable, our findings demonstrate possible characteristics that are specific to multiple intracranial aneurysms. The findings of our research remain to be further verified in larger cohorts from multiple centers. Like other retrospective studies, the images of the ruptured aneurysms may have been affected by the event of the rupture itself. Even though prior evidence indicates that such a change does not occur, consistent with intraoperative findings, conclusive data on this subject remain to be found [37]. In addition, the mechanisms of multiple intracranial aneurysms rupture cannot be elucidated by morphology and/or hemodynamics. The hemodynamics effects on the endothelial cell are still unclear, since the endothelial lining was found to be absent in 30% of the unruptured aneurysms and 62% of ruptured aneurysms [38]. It is necessary to assess the hemodynamic impact on the aneurysm wall without endothelial cells in the future.

## Conclusions

Using a study model in which we compared ruptured and unruptured aneurysms in the same patients harboring multiple intracranial aneurysms, a detailed analysis of morphological and hemodynamic features was evaluated. This might help us to understand the mechanisms of multiple intracranial aneurysms rupture and assess the rupture risk of unruptured aneurysms. Large aspect ratio and low WSSmean were independently associated with the rupture status of multiple intracranial aneurysms. These findings in multiple intracranial aneurysms need to be further confirmed based on large multicenter and multipopulation data.

## Supporting Information

S1 Table Characteristics of patient population and data used for the morphologic and hemodynamic analysis. (XLSX)
